# Triptolide targets super-enhancer networks in pancreatic cancer cells and cancer-associated fibroblasts

**DOI:** 10.1038/s41389-020-00285-9

**Published:** 2020-11-09

**Authors:** Pawan Noel, Shaimaa Hussein, Serina Ng, Corina E. Antal, Wei Lin, Emily Rodela, Priscilla Delgado, Sanna Naveed, Michael Downes, Yin Lin, Ronald M. Evans, Daniel D. Von Hoff, Haiyong Han

**Affiliations:** 1 Molecular Medicine Division, Translational Genomics Research Institute, Phoenix, AZ USA; 2 Baylor Scott and White Research Institute, Baylor Institute for Immunology Research, Dallas, TX USA; 3 Gene Expression Laboratory, Salk Institute for Biological Studies, San Diego, CA USA; 4 Howard Hughes Medical Institute, Salk Institute for Biological Studies, San Diego, CA USA; 5 Present Address: Phoenix Children’s Hospital, Phoenix, AZ USA

**Keywords:** Pancreatic cancer, Target validation

## Abstract

The tumor microenvironment in pancreatic ductal adenocarcinoma (PDAC) is highly heterogeneous, fibrotic, and hypovascular, marked by extensive desmoplasia and maintained by the tumor cells, cancer-associated fibroblasts (CAFs) and other stromal cells. There is an urgent need to identify and develop treatment strategies that not only target the tumor cells but can also modulate the stromal cells. A growing number of studies implicate the role of regulatory DNA elements called super-enhancers (SE) in maintaining cell-type-specific gene expression networks in both normal and cancer cells. Using chromatin activation marks, we first mapped SE networks in pancreatic CAFs and epithelial tumor cells and found them to have distinct SE profiles. Next, we explored the role of triptolide (TPL), a natural compound with antitumor activity, in the context of modulating cell-type-specific SE signatures in PDAC. We found that TPL, cytotoxic to both pancreatic tumor cells and CAFs, disrupted SEs in a manner that resulted in the downregulation of SE-associated genes (e.g., BRD4, MYC, RNA Pol II, and Collagen 1) in both cell types at mRNA and protein levels. Our observations suggest that TPL acts as a SE interactive agent and may elicit its antitumor activity through SE disruption to re-program cellular cross talk and signaling in PDAC. Based on our findings, epigenetic reprogramming of transcriptional regulation using SE modulating compounds such as TPL may provide means for effective treatment options for pancreatic cancer patients.

## Introduction

Pancreatic cancer (PC), currently the third leading cause of cancer-related death in the United States^1^, is projected to be the second leading cause of cancer-related death before 2030^2^. Pancreatic ductal adenocarcinoma (PDAC) accounts for >90% of all PC cases and has a dismal 5-year survival rate of 9.3%, mainly due to the lack of reliable methods for early detection and limited treatment options^3^. A unique, highly fibrotic, and hypovascularized tumor stroma or “desmoplastic reaction” (DR) forms a major barrier to currently available therapeutics^4^. Cancer-associated fibroblasts (CAFs), one of the major cell types in the PDAC tumor microenvironment (TME), play an important role in driving and maintaining the DR^5^. Although recent genomic analysis reveals the underlying genetic diversity of PDAC^6^, the almost uniformly inflammatory and immunosuppressive stroma in pancreatic cancer suggests common mechanisms that drive its progression and metastasis^7^. Thus, therapeutic regimens that re-program (or modulate) stromal elements, in addition to targeting the tumor cells, may potentially enhance the efficacy of antitumor and immunotherapeutic agents, which can eventually improve outcomes for patients with PC.

With advancements in global genomic analyses, new insights into the transcriptional regulation of gene expression implicate the role of super-enhancers (SE) in maintaining cell-type-specific gene expression networks in both normal and diseased cells^8,9^. SEs are transcriptional regulatory elements composed of regular enhancers clustered together in close genomic proximity and bear active chromatin marks such as H3K27 acetylation (H3K27ac) and BRD4 occupancy. In addition, SEs are associated with accessible chromatin regions, DNase hypersensitivity, RNA Pol II loading, and extensive binding of transcription factors and mediator proteins, specifically Med1^9^. Since the first reports of SEs in human embryonic stem cells, there is mounting evidence for their role in regulating cell identity and state in numerous diseases including cancer, where they promote oncogenic transcriptional dependencies^10,11^. Recently, inhibitors of CDK7, a subunit of the transcription factor complex TFIIH, were shown to disrupt SE networks in cancer cells and exert potent antitumor activity^12–14^. However, the effects of other TFIIH-targeted molecules have not been reported.

Triptolide (TPL), a natural compound isolated from the Chinese herb *Tripterygium wilfordii* (Thunder God vine), has shown promising preclinical antitumor activity against a number of cancers^15–17^ including pancreatic cancer^18–20^. The water-soluble pro-drug of triptolide, Minnelide, was recently reported to show promising activity in patients with gastrointestinal malignancies in Phase I clinical trial^21,22^. Multiple mechanisms underlying TPL-induced antitumor activity have been described in the literature including inhibition of NFκB, c-Myc, HSP70^23–30^, and XPB (ERCC3)^31^. Among them, inhibition of the ATPase activity of the XPB subunit of the transcription factor complex TFIIH is supported by biochemical evidence, which shows direct covalent binding of triptolide to XPB^32^. However, it is unclear how this seemingly non-specific inhibition of an essential transcription factor could exert selectivity against the tumor. This work focused on elucidating the impact of triptolide on the SE networks in pancreatic tumor cells and CAFs. Here, we profiled the SE networks in pancreatic CAFs and epithelial tumor cells and examined the effects of triptolide on the SE signatures of both cell types.

## Materials and methods

### ChIP-Seq analysis and super-enhancer comparison

ChIP-Seq reads were mapped to the human reference genome (hg19) using Bowtie2 with default parameters. H3K27Ac peak calling was performed using the Model-based Analysis of ChIP-Seq (MACS) program (version 1.4.2)^33^ with default settings. Peak calling for each sample was performed separately with their matched input genomic DNA as a background control. The enrichment and ranking of super-enhancer regions and identifying SE-associated genes was performed using ROSE^9,34^. ROSE-assigned closest genes to the SEs were used as the default list for SE-associated genes. H3K27Ac peaks that fell within the region surrounding ±2.5 kb of the transcription start site (TSS) were considered as promoter peaks and excluded from the super-enhancer analysis. Calling of ChIP peaks and differential signals were also determined using HOMER modules on the Linux platform^35^. BedGraph files representing the mapped read counts of individual samples were also generated using Homer and uploaded to the UCSC genome browser for display. HOMER was also used to generate the scatter plot comparing SE differential peaks in treated vs untreated samples using annotatePeaks command with size 2000 and the log options.

### Whole transcriptome RNA-sequencing (RNA-seq) and data analysis

The RNA-seq analysis was performed as previously described^36^. RNA extraction was performed using the RNeasy^®^ Midi Kit (Qiagen) using protocols recommended by the kit manufacturer. RNA-sequencing libraries were constructed using the NEB Next^®^ Ultra™ RNA Library Prep Kit (New England Biolabs) by Novogene. The concentration of libraries for RNA-seq from total RNA was first quantified using a Qubit 2.0 fluorometer (Life Technologies), and then diluted to 1 ng/μl before checking insert size on an Agilent 2100 and quantifying to greater accuracy by quantitative PCR (Q-PCR) (library activity >2 nM). Libraries were sequenced on an Illumina^®^ HiSeq2500 system.

Raw paired-end reads from the sequencer were then processed to obtained FPKM (fragments per kilobase of transcript per million) mapped reads (see Supplementary Information for details). Different gene expression analysis was carried out using the DESeq2 R package. To compare the overall transcription activity between two samples, normalized counts for each sample were calculated using DESeq2, which performs an internal normalization where geometric mean is calculated for each gene across all samples. The counts for a gene in each sample was then divided by this mean. This procedure corrects for library size and RNA composition bias, which can arise for example when only a small number of genes are very highly expressed in one experiment condition but not in the other^37^.

### Animal studies

All animal studies were carried out adhering to recommendations in the NIH Guide for the Care and Use of Laboratory Animals. The protocols were approved by the Institutional Animal Care and Use Committee (IACUC) at the University of Arizona, where the animal studies were carried out.

Transgenic KPC (LSL-KrasG12D/+; LSL-Trp53R172H/+; Pdx-1-Cre) mice were obtained based on the breeding scheme described by Hingorani and colleagues using three mouse strains, LSL-Trp53R172H/+, LSL-KrasG12D/+, and Pdx-1-Cre, which were obtained from National Cancer Institute Mouse Repository^38^. Mice were fed ad libitum and housed at ambient temperatures (70–76 °F). Tumor growth in the KPC mice was monitored using three-dimensional high-resolution ultrasonography with the Visualsonics Vevo 770 system (Fujifilm Visualsonics, Ontario, Canada). Mice (both male and female) were enrolled in the study when the tumor size reached 120–200 mm^3^. Mice (*n* = 5) were treated with Minnelide via i.p. at 0.42 mg/kg daily for 7 days, after which tumor volumes were recorded and tumors were harvested. Total RNA was extracted from representative tumor pieces for RNA-sequencing (*n* = 3) and immunostaining analyses (*n* = 3).

The in vivo tumor growth inhibition activity of Minnelide alone or in combination with chemotherapy was evaluated using patient-derived xenograft (PDX) models derived from two different PDAC patients (U01080713 and P4057). Detailed methods for drug treatment and endpoint measurements can be found in the Supplementary Information.

### Sample size calculation

In the animal study with the KPC mouse model, the mean difference in tumor volume between the vehicle-treated group (298.5 ± 45.2) and the Minnelide-treated group (202.1 ± 28.1) was 96.3 mm^3^. To detect this difference with 90% power, we need three mice per group (*α* = 0.05). With five mice per group, our study is sufficiently powered. Similarly, for the studies with the PDX models, the differences in tumor volume between the monotherapy (Minnelide only or triple chemotherapy only) and the combination treatment were 165 mm^3^ and 127 mm^3^ for PDX U01801713 and PDX P4057, respectively. To detect these differences between the monotherapies and the combination therapy with the typical variations associated with PDX models, we needed five mice per group to achieve 90% detection power (*α* = 0.05). With seven mice per group, our studies were sufficiently powered.

### Data analysis and statistical methods

The statistical analysis for the cytotoxicity assay was carried out using GraphPad Prism Software (GraphPad, San Diego, CA, USA) to plot the curves for each cell line. Statistical significance for the IHC staining between vehicle and Minnelide-treated groups was evaluated using a two-tailed Wilcoxon Rank Sum non-parametric test. The mixed-effects model analysis was used to evaluate the statistical differences between two drug treatment groups over a period of time^39^ in the mouse efficacy study. A *P*-value < 0.05 was considered to represent statistical significance.

## Results

### Pancreatic tumor cells and cancer-associated fibroblasts have distinct SE profiles

**Fig. 1:**
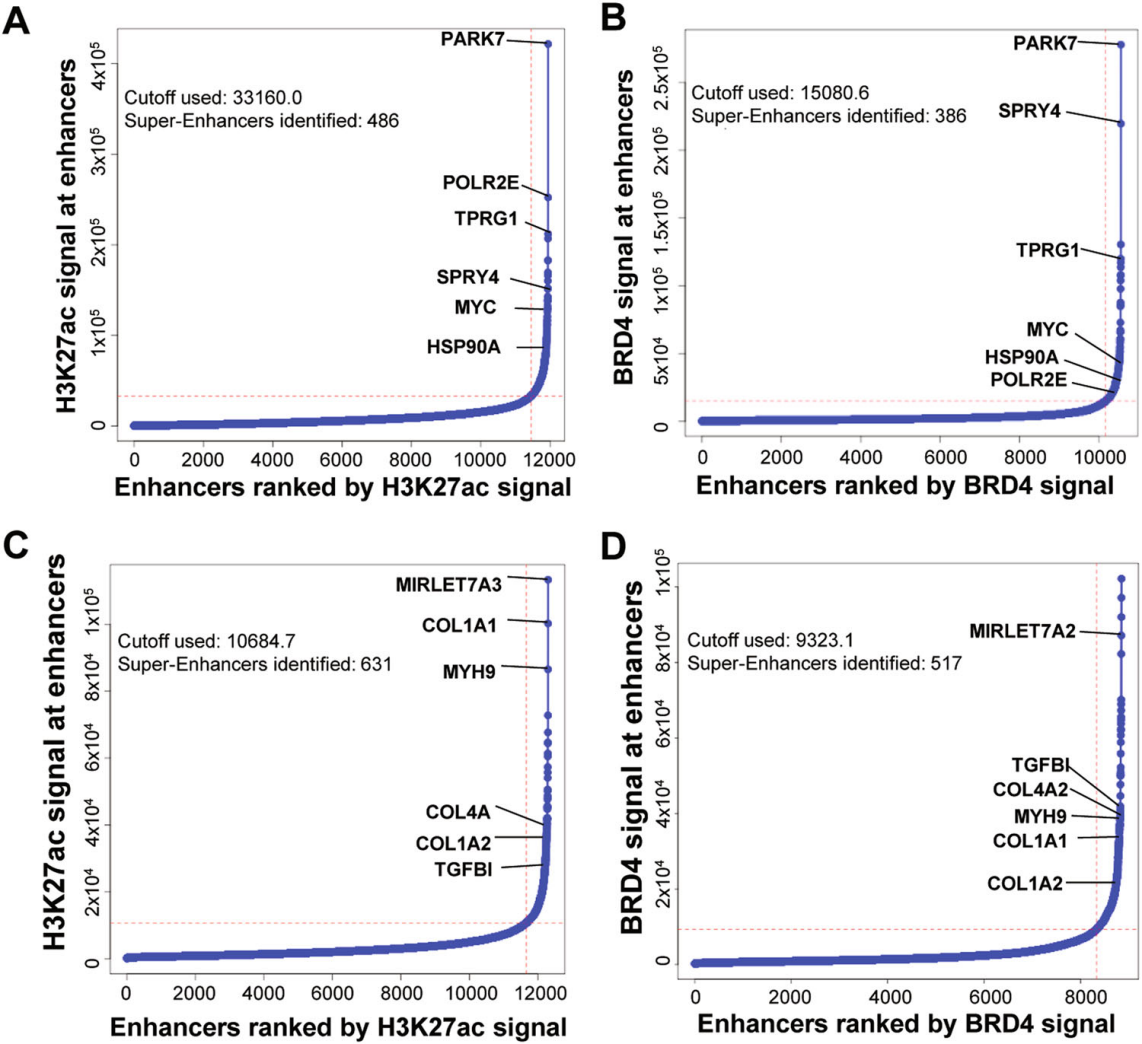
Super-enhancers identified in the MIA PaCa-2 and CW5 cell lines. **A**–**D** Hockey plots showing enhancer ranking based on the distribution of both H3K27ac signals (**A**, **C**) and BRD4 occupancy (**B**, **D**) in MIA PaCa-2 (**A**, **B**) and CW5 cells (**C**, **D**). Super-enhancers are seen above the inflection point of the curve. Distinct SEs revealed in each cell line show a remarkable overlap between H3K27ac and BRD4-based rankings in each cell line.

Using chromatin immunoprecipitation followed by high throughput sequencing (ChIP-seq), we mapped genomic loci that represent active super-enhancer (SE) elements. We carried out SE mapping in a panel of PDAC cell line (MIA PaCa-2, PANC-1, P4057, PSN1, AsPC1, Capan-1, BxPC-3, PA-TU8902, and PA-TU8988S), and in a panel of CAFs isolated from a PDAC patient’s tumor (CW5, CAF08, CW1, B010A, B010C, B009B, and BF1) along with an activated immortalized pancreatic stellate cell line (PS-1). We performed ChIP-Seq analyses using well-known marks for active SEs, acetylation of histone 3 at lysine 27 (H3K27ac)^40^. For two of the cell lines (MIA PaCa-2 and CW5), we also performed ChIP-Seq analysis with a second SE mark, BRD4. Figure 1 shows the hockey-stick plots of SEs ranked based on both these chromatin marks. Many of the ChIP signals overlapped between the H3K27ac and BRD4 marks, which supports the notion that these elements are active SEs in the cell lines. A similar number of SE elements in two cell lines was identified (318 SEs in MIA PaCa-2 tumor cells and 337 SEs in CW5 CAF cells). Genes associated with the SEs in tumor cells were mostly related to transcription (e.g., *POLR2E, PARK7, MYC*) (Fig. 1a, b). PARK7 and MYC have both been reported previously to be associated with PDAC^41–43^. On the other hand, SE-associated genes in CAFs were mostly associated with processes such as desmoplasia, fibrosis, or those related to the extracellular matrix (e.g., *COL1A1, COL1A2, TGFBI*), which is abundant in advanced-stage PDAC (Fig. 1c, d).

**Fig. 2:**
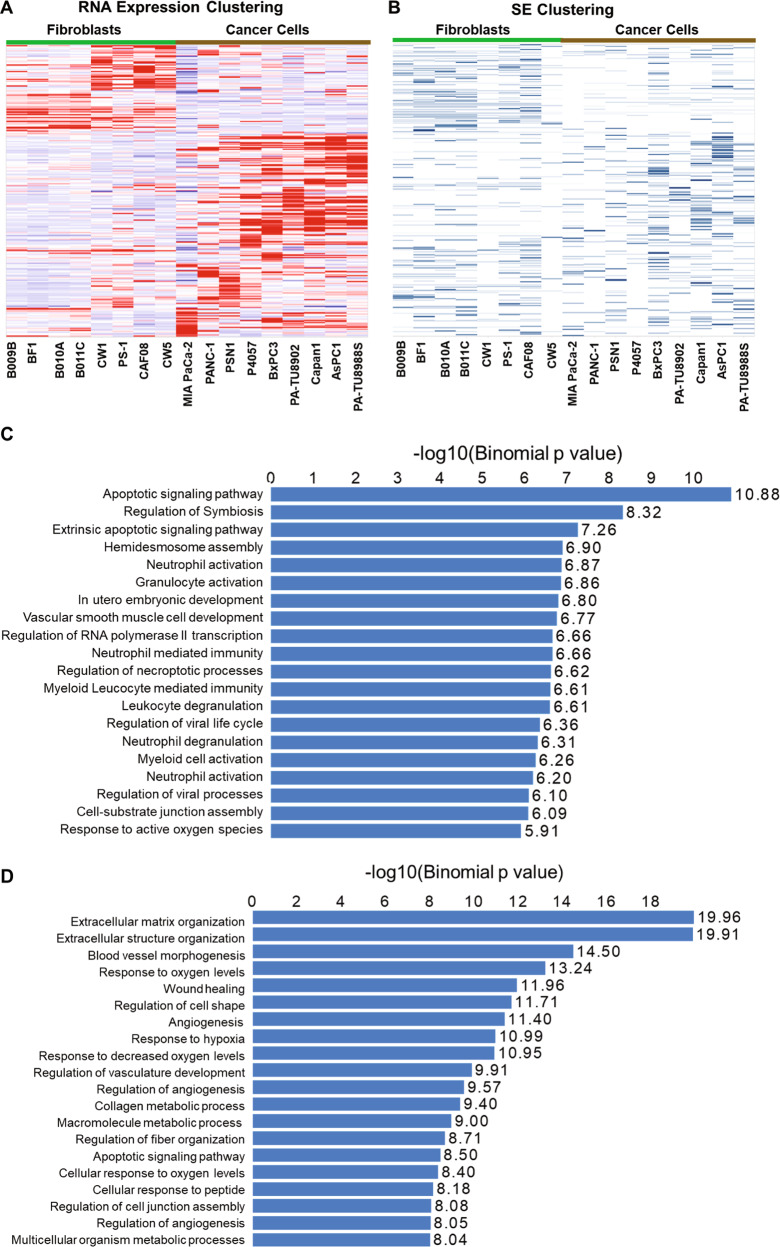
Pancreatic tumor cells and cancer-associated fibroblasts harbor distinct super-enhancer networks. **A** Heatmap for the hierarchically clustered expression data of SE-associated genes identified in the pancreatic cancer cell lines and CAF cells. PS-1 is an activated immortalized pancreatic stellate cell line. **B** Heatmap representing all SE-associated genes as identified by ROSE, clustered according to panel **A** to show the presence (blue) or absence (white) of a SE. **C** Gene Ontology (GO) biological processes enriched in super-enhancer regions in PDAC cancer cell lines. **D** GO biological processes enriched in super-enhancer regions in fibroblast cell lines. The *P*-value scores [shown as −log (*P*)] and significance directly correlate with the number of genes contained in each GO term. Each GO term containing more genes tends to have a higher significance *P*-value.

We then analyzed the SEs that were identified based on the H3K27ac ChIP-Seq profiling of the two cell line panels to see whether the cancer cells and the CAF cells demonstrate similar distinct patterns. A total of 3452 SEs were identified from the cell lines (SEs being called in at least one cell line) (Supplementary Table S1). Based on the Homer program, we identified the genes that were associated with these SEs and analyzed their expression using RNA-seq (Supplementary Table S2). Hierarchical-clustering analysis of the expression of the SE-associated genes showed that cancer cells and CAFs form clearly separate clusters (Fig. 2a). The pattern of expression of these genes in different cell lines closely mirrors whether or not the corresponding SE is active in the given cell line (Fig. 2b). The immortalized and activated pancreatic stellate cell line PS-1^44^ also clustered with the CAF lines indicating their similar SE profiles (Fig. 2).

The distinct nature of SE signatures in two predominant cell types in pancreatic tumors led us to investigate the nature of these signatures and to unravel signaling and cellular pathways they may be associated with. For this, we carried out genomic region annotation enrichment analysis on SE regions that are found in at least 3 of either the cancer cell lines or fibroblast cell lines using a web-based analysis tool GREAT (see Materials and Methods for details). As shown in Fig. 2c, d and Supplementary Fig. S1A, B, SEs from the two cell types enriched for different functional annotations (Gene Ontology terms). Cancer cell-associated SEs networks (Fig. 2c) seem to be enriched in biological processes regulating transcription, apoptosis, and immune function. On the other hand, SE-associated gene networks in CAFs (Fig. 2d) span biological processes involved in an extracellular matrix organization, angiogenesis, and hypoxia. Interestingly, both cell types contained SEs that were associated with molecular functions related to cell adhesion (Supplementary Fig. S1). Our observations shed light on the diversity and cell-type specificity of the SE signatures within the pancreatic cancer TME.

### Triptolide suppresses super-enhancer activation marks and causes transcriptional downregulation of associated genes in pancreatic cancer cells and CAFs

We first assessed the optimal inhibitory concentrations of TPL for use in in vitro studies in multiple cell lines (Supplementary Fig. S2). Based on these results, we determined the optimal concentrations of TPL to use for each cell line while performing genomic and transcriptomic assays discussed below.

**Fig. 3:**
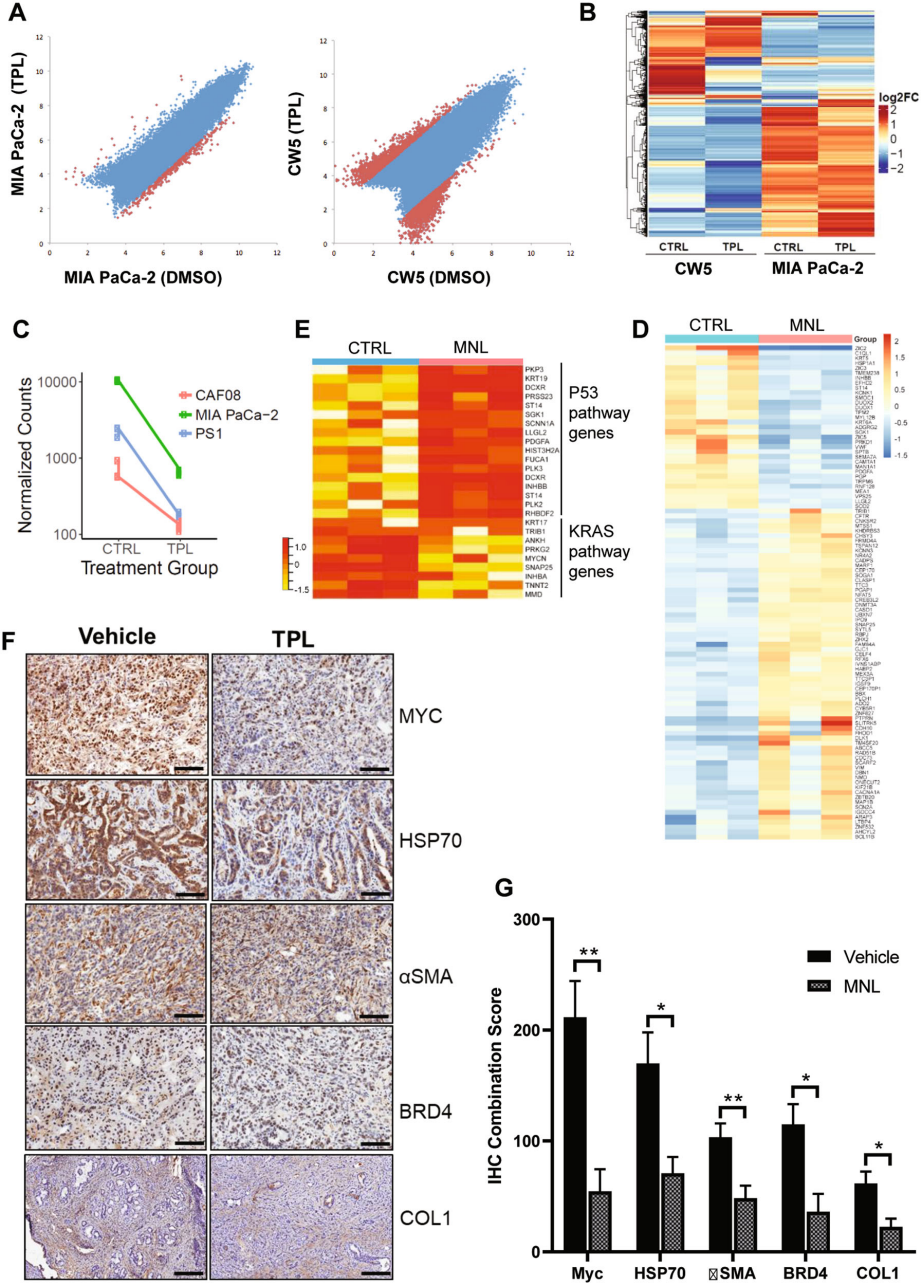
Triptolide downregulates H3K27ac chromatin marks, overall in vitro gene transcription, and causes deregulation of cancer pathways in animal models. **A** Scatter plots of H3K27ac ChIP peak signals are shown for tumor (MIA PaCa-2) and CAF (CW5) cells, showing a substantial downregulation of peaks in triptolide (TPL) treated cells. Notably, CW5 show a larger reduction of peaks compared to tumor cells. **B** Hierarchical-clustering of differentially expressed genes in CW5 and MIA PaCa-2 cells in response to TPL is shown as a heatmap. The overall reduction in gene expression expressed as log2 fold change is noted in TPL-treated cells compared to DMSO-treated control cells (CTRL). **C** The effect of TPL in inducing downregulation of gene expression (expressed as normalized counts) as seen in tumor cells (MIA PaCa-2) and fibroblasts (CAF08, PS-1) is shown. **D** Heatmap of the top 100 differentially expressed genes in pancreatic tumors harvested from the transgenic mouse model of pancreatic cancer (KPC mice) upon Minnelide (MNL) treatment (*n* = 3 for each treatment group). **E** Heatmap of gene sets belonging to TP53 and KRAS pathways that are differentially regulated in the Minnelide-treated mouse tumors. **F** Minnelide reduces the expression of SE-associated genes in mice bearing pancreatic tumors. Representative immunostaining images of SE-associated gene markers (HSP70, MYC, αSMA, BRD4, and COL1) in control and Minnelide-treated samples. Scale bar = 100 µM. **G** Quantification of immunostaining in **F** (*n* = 3). *P < 0.05; **P < 0.01. Error bars represent the standard error of the mean (SEM). TPL Triptolide, MNL Minnelide.

To evaluate the effects of triptolide on well-known chromatin activation marks of SEs in cells, we compared the intensity of the H3K27ac immunoprecipitation signals before and after treatment in the MIA PaCa-2 cancer cells and the CW5 CAF cells. ChIP-Seq peaks with a >4-fold change in H3K27ac signal were considered significantly altered. At this threshold, we noticed a substantial difference in the H3K27ac signals between TPL- and vehicle- (DMSO) treated tumor cells and CAFs (Fig. 3a). While triptolide caused a marked reduction in the number of H3K27 acetylation peaks in both cell lines, a more pronounced effect was elicited in CW5 compared with that that in MIA PaCa-2. H3K27ac signals at some gene-associated loci seemed to be increased in CW5, with a slightly higher number of loci showed a decrease in the SE activation mark. In contrast to CW5 CAF cells, about a tenth of the loci showed a decrease in signal in MIA PaCa-2 tumor cells. Results from the analysis of differential gene expression in response to TPL treatment in MIA PaCa-2 and CW5 cells (Fig. 3b) showed similar patterns of larger changes in the CAFs versus the tumor cells. We also tested the overall effect of TPL on the transcriptomes of CAF08 and PS-1 cells. Similar to that of CW5 cells, TPL induced greater changes in those stromal cells compared to MIA PaCa-2 cancer cells. TPL causes overall transcriptional downregulation of gene expression in all three cell lines as seen by a decrease in the normalized counts for differential gene expression (Fig. 3c).

Next, we examined the effects of TPL on SE-associated genes in mouse PDAC tissues using the KPC pancreatic cancer mouse model. TPL is not very water-soluble and is not optimal for in vivo experiments. We, therefore, used Minnelide, a water-soluble pro-drug of triptolide, for animal studies in this study. Minnelide is rapidly converted to triptolide in the presence of alkaline phosphatase in the body (half-life = 2 minutes) and has been shown to have potent antitumor activity as a single agent in animal models for pancreatic cancer^19^. As shown in Supplementary Fig. S3, the daily dosing of Minnelide at 0.42 mg/kg significantly (*P*-value < 0.004) reduced the tumor growth in the KPC mice. RNA-seq analysis of the PDAC tumor tissues harvested from Minnelide-treated mice clearly revealed its effect in deregulating gene expression (Fig. 3d). In the same mouse tumors, we noticed that genes related to the TP53 pathway were upregulated by Minnelide, while those involved in the KRAS hallmark pathway seem to be inhibited (Fig. 3e). Immunohistochemical analysis of the tumor tissues revealed a statistically significant (*P*-value < 0.05) reduction in staining for HSP70, MYC, BRD4, and αSMA (Fig. 3f, g) in Minnelide-treated mice. This suggests that Minnelide downregulated the protein expression of these SE genes in mouse pancreatic tumors.

### Discrete SE targets in tumor and CAF cells are specifically disrupted by triptolide

**Fig. 4:**
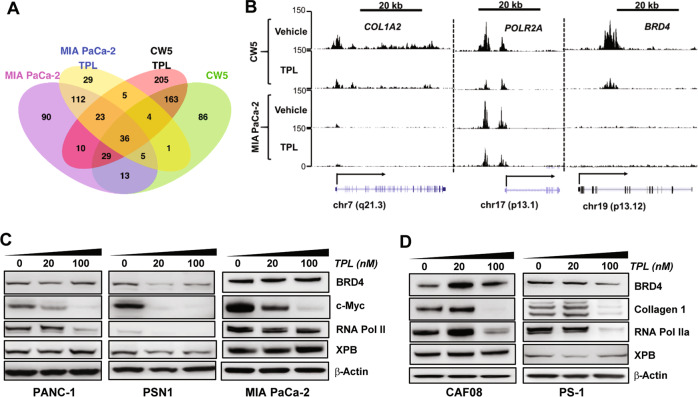
Triptolide disrupts cell-type-specific SEs by reducing chromatin activation marks and resulting in the downregulation of SE-associated genes. **A** Venn diagram summarizing SEs identified in tumor and fibroblast cells and those identified in TPL-treated cells. **B** Gene tracks of H3K27Ac-binding density at SEs associated with POL2RA, COL1A2, and BRD4 genes in patient-derived CAF cells (CW5, top 2 panels) and MIA PaCa-2 cancer cells (bottom 2 panels) treated with DMSO (Vehicle) or 100 nM triptolide (TPL). **C**, **D** Triptolide downregulates the expression of genes under SE regulation at the protein level. Western blotting was used to probe the protein levels of SE-associated genes (*BRD4*, RNA Pol II, Collagen 1) and non-SE-associated gene XPB in 3 cancer cell lines (PANC-1, PSN1, MIA PaCa-2) (**E**) and activated fibroblast cells (CAF08 and PS-1) (**F**) treated vehicle, 20 nM, or 100 nM of TPL for 24 h. *P < 0.005; **P < 0.0001.

To further dissect the effects of triptolide on SEs, we focused on cell-type-specific SEs and those that were commonly identified in both cancer and CAF cells. First, we identified genes that were associated with SEs, inferred from the ROSE algorithm based on the relative proximity of the gene to a SE element. Several genes could be under the influence of a single SE based on enhancer/chromatin looping. We identified 315 and 337 SEs in the vehicle-treated MIA PaCa-2 and CW5 cells, respectively. While about 30% of the genes associated with these SEs were common between the two cell lines, a larger proportion (70%) were uniquely identified in each cell line (Fig. 4a). We compared the SE-associated genes in both cell lines with and without triptolide treatment. The number of SE-associated genes in TPL-treated MIA PaCa-2 and CW5 cells was 215 and 475, respectively. Our observations indicate that around 56% of the SE-associated genes do not overlap between the control and TPL-treated groups in MIA PaCa-2 cells, while the percentage of non-overlapping genes was close to 69% in CW5 cells. This is consistent with our findings that TPL had a greater impact on SEs in the CW5 cells than the MIA PaCa-2 cells (Fig. 3a). Overall, these observations suggest that TPL disrupts SE elements in both PDAC tumor cells and CAFs.

We further investigated the effect of triptolide on the distinct SE activation marks mapped to individual cell lines. To this end, we compared ChIP signals in the vehicle and TPL-treated cells at loci representing SE elements in one or both of the cell lines. In CW5 cells, SEs associated with POL2RA, COL1A2, and BRD4 loci exhibited a decrease in the H3K27ac mark for SE activation upon TPL treatment (Fig. 4b). On the other hand, in MIA PaCa-2 cells, TPL induced a marked decrease in H3K27ac marks at the POL2RA locus, but not at COL1A2 and BRD4. The latter two loci were not identified as SEs in MIA PaCa-2 cells. This observation supports the notion that TPL acts on and disrupts distinct cell line-specific SEs to modulate the expression of the associated genes.

### SE-associated genes are downregulated by TPL at the protein level

To further validate the effects of TPL on the expression of SE-associated genes, we investigated the expression of candidate SE genes at the protein level in multiple pancreatic cancer and activated fibroblast cell lines. We performed immunoblotting in vehicle and TPL-treated pancreatic cancer cells (PANC-1, PSN1, MIA PaCa-2) and in fibroblast cells (CAF08, PS-1). TPL treatment (20 nM and 100 nM for 24 h) reduced the protein expression levels of BRD4, MYC, and RNA Pol II in the tumor cell lines (Fig. 4c). In fibroblast cells, protein levels of BRD4, RNA Pol II, and CAF-specific/SE-driven COL1A2 (Collagen 1) did not change at 20 nM but were considerably reduced at 100 nM in both CAF08 and PS-1 fibroblasts cells (Fig. 4d). We also looked at the expression of a non-SE-associated gene, *XPB* (a direct target of TPL), and did not notice appreciable changes in its expression in both tumor and CAF cells (Fig. 4c, d). This was somewhat expected and used as a control since TPL only inhibits the ATPase activity of XPB but does not affect its expression levels^20^.

### Targeting of super-enhancers by triptolide is similar to that by CDK7 inhibition

**Fig. 5:**
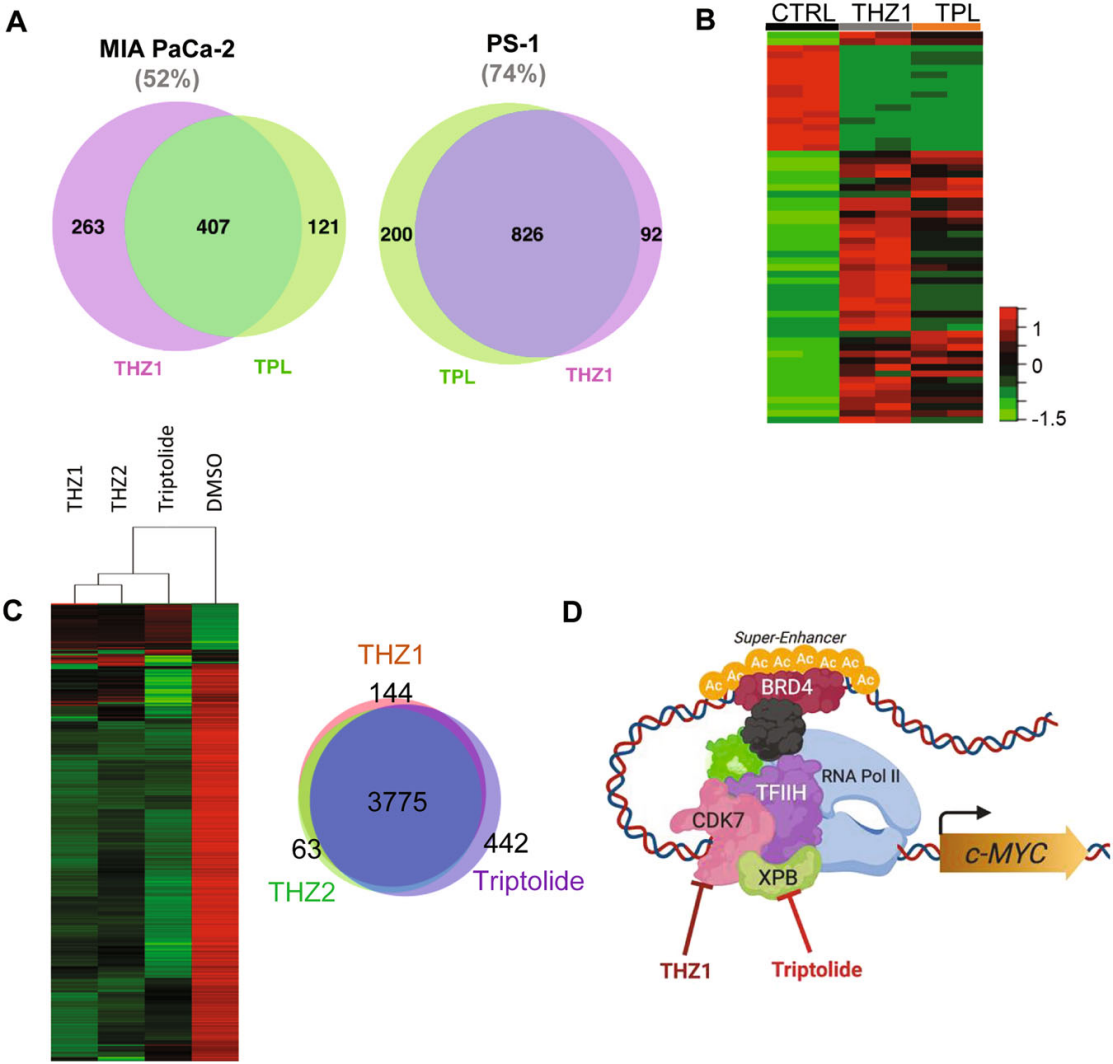
Triptolide targets SE networks similar to that induced by CDK7 inhibition. **A** Overlap of SE-associated genes in MIA PaCa-2 and PS-1 cells treated with TPL or CDK7 inhibitor THZ1. **B** Clustering analysis of gene expression profiles of MIA PaCa-2 cells treated with TPL and THZ1. **C** Clustering analysis of gene expression profiles of PANC-1 cells treated with various epigenetic inhibitors including TPL, THZ1, and THZ2. **D** A model for the mechanism of action of TPL and CDK7 inhibitors. Inhibition of one of the components (e.g., XPB and CDK7) of the transcription machinery leads to the disruption of the activity of a SE, which in turn results in the suppression of the gene associated with that SE.

Recent reports show that THZ1, a CDK7 inhibitor, suppresses SE-associated oncogenic transcription in MYC-deregulated cancer models^12,14^. Here, we compared the SE networks and transcriptional effects of TPL to those of THZ1. Interestingly, we noticed that both THZ1 and TPL, which inhibit the CDK7 and XPB subunits of the TFIIH complex, respectively, targeted SEs and SE-associated genes similarly. In terms of SE, we identified considerable overlaps between SE regions in TPL- and THZ1-treated tumor (MIA PaCa-2) and fibroblast (PS-1) cells. We noticed that 52% of SE regions were common to both treatments in MIA PaCa-2 cells and an even higher 74% of the SE regions were shared between TPL- and THZ1-treated PS-1 cells (Fig. 5a). This result supports the proposed mechanism of the two compounds (i.e., inhibition of TFIIH activity), leading to the disruption of global super-enhancer activity. When comparing the expression changes of SE-associated genes in MIA PaCa-2 cells treated with TPL or THZ1, a high degree of similarity between the transcriptional profiles in both treatment groups was noticed (Fig. 5b). RNA-Seq analysis interrogating the transcriptional effects of TPL, THZ1, and another CKD7 inhibitor THZ2, in a different PDAC cell line, PANC-1, showed similar gene expression changes induced by all three compounds. THZ2 showed an over 85% similarity in gene expression profiles in PANC-1 cells (Fig. 5c). Collectively, these findings suggest that the anti-SE activity of TPL is a result of its inhibition of XPB, which leads to the disruption of TFIIH activity as in the case with CDK7 inhibitors. Thus, cumulative pressure on the transcriptional machinery using BET inhibitors, CDK7 inhibition, and TPL may hold greater potential in global disruption of SE elements in tumor cells (Fig. 5d).

### Combination of Minnelide and chemotherapy show improved antitumor activity in vivo

**Fig. 6:**
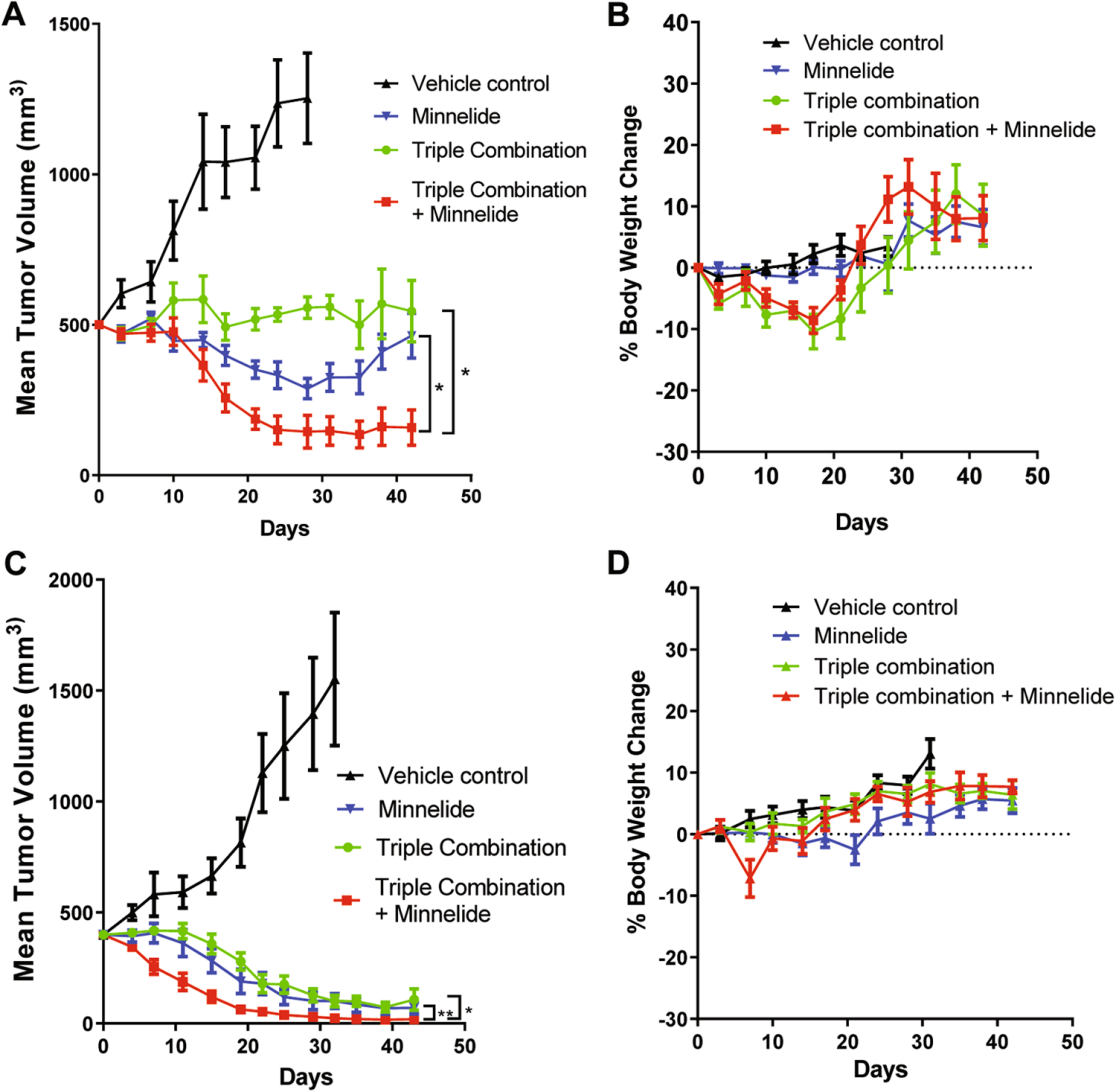
Minnelide synergizes with chemotherapy in suppressing tumor growth in PDX models for pancreatic cancer. **A** Tumor growth curves in the U01080713 PDX model. **B** Mouse body weight changes during the treatment for the P4057 model. **C** Tumor growth curves in the P4057 model. **D** Mouse body weight changes during the treatment for the P4057 model. *P-value < 0.0001; **P-value = 0.0002 by mixed-effects model.

As mentioned earlier, Minnelide is a water-soluble pro-drug of triptolide with potent single-agent activity in mouse models of PDAC^26^. To test whether Minnelide can enhance the antitumor activity of chemotherapeutics, we carried out animal studies using two different patient-derived xenograft (PDX) models. In both models, treatment with either Minnelide or a triple chemotherapy combination (gemcitabine, nab-paclitaxel, and cisplatin) showed very impressive tumor regression (Fig. 6). Treatment with Minnelide alone led to tumor shrinkage in both models, whereas the triple chemotherapy showed complete suppression of tumor growth in one model (Fig. 6a) and slight tumor shrinkage in the other model (Fig. 6c). When the two treatments were combined, the tumor shrinkage was marked in both models. Adding Minnelide to the triple chemotherapy regimen did not seem to increase toxicity (Fig. 6b–d). The triple chemotherapy has recently been reported to show promising efficacy in patients with advanced PDAC with a 71% response rate and a 65% 1-year survival rate for patients with Stage IV pancreatic cancer with some 3- and 4-year survivors^45^. Our results show that adding Minnelide to the combination therapy could further improve patient outcomes. This finding awaits further validation by a clinical trial of that combination (given concurrently or sequentially).

## Discussion

Despite the genetic diversity in PDAC, the commonality in the mutational and phenotypic signatures of pancreatic cancer point to master regulatory networks gone awry in this devastating illness^7^. This may provide immense therapeutic potential in targeting oncogenic networks that drive cellular and molecular cross talk in pancreatic tumors through the employment of agents that can disrupt, modulate, or re-engineer these mechanisms. In this study, we provide evidence supporting the presence of distinct super-enhancer signatures defining the two predominant cell types in the cellular milieu of PDAC. By carrying out genomic analyses and mapping super-enhancers in both CAFs and tumor cells, we provide insights into the epigenetic landscape that shapes and drives tumor supportive oncogenic networks, which may eventually mediate cellular cross talk in PDAC. The largely distinct cell-type-specific nature of SEs allows the investigation of altered pathways that may be modulated in different compartments of the pancreas TME to disrupt oncogenic signaling promoting tumor growth, proliferation, and invasion.

The major theme that emanates from this study is the effect of triptolide on SE elements per se and on the downstream effector gene(s) associated with SE elements. While triptolide (TPL) has been known to be a general transcriptional inhibitor and a potent antitumor agent acting predominantly via the inhibition of MYC, NFκB, and other pathways to induce cell death^24,25,27,46,47^, we are the first to report its role in modulating SEs at the chromatin level to downregulate mRNA and protein expression of genes under SE control. One of the most critical molecular players involved in diverse cellular signaling pathways in many cancers including pancreatic cancer is the MYC proto-oncogene^48,49^. MYC is overexpressed in pancreatic cancer^50^ where it acts as a transcription factor^51^ and is also implicated in determining phenotypic plasticity of pancreatic cancer stem cells^52^. Our study supports MYC to be a SE-associated gene in pancreatic cancer cells, as also reported by other groups^7^. TPL induced downregulation of MYC in a dose-dependent manner in multiple pancreatic tumor cell lines that we tested, suggesting that it is one of the major targets of triptolide. While the use of direct and indirect targeting of MYC in pancreatic cancer is under development^53^, targeting SE-regulated gene networks such as those like MYC with TPL can lead to the elucidation of novel vulnerabilities, which may be eventually targeted using combinatorial therapeutic strategies. The disruption of the SEs regulating collagen expression in CAF cells in response to triptolide has profound implications, since strategies like these which modulate and compromise the supporting tumor stroma in PDAC may increase therapeutic penetrance and immune infiltration. Clinically, Minnelide, a water-soluble form of triptolide, has shown promising activity in pancreatic and gastric cancers^21,22^ and is currently in Phase II clinical trials for patients with refractory pancreatic cancer (NCT03117920).

The feasibility of disrupting SEs is in development using various agents such as those that target CDK7^12,14^ and BRD4 proteins^34,54^. TPL-mediated disruption of cell-specific SEs to downregulate gene expression (*MYC, BRD4*, RNA Pol II, *COL1A2*, etc.), as elucidated here, implies its therapeutic relevance in targeting both stromal and tumor compartments of PDAC. Interestingly, our data suggest that TPL elicits a similar transcriptional change to that of CDK7 inhibition by THZ1 or THZ2 in pancreatic cancer cells. Furthermore, a recent study implicates XPB as a direct TPL target, which along with CDK7 and other factors forms part of the TFIIH transcriptional complex to drive constitutive transcription of genes^31^. It is likely that similar to CDK7 inhibitors, the inhibition of XPB activity by TPL can disrupt the interactions between SE and transcription factors/co-activators, which, in turn, leads to the dysregulation of genes associated with the SEs. Interestingly, XPB was recently shown to interact with MYC in patient-derived xenograft models of PDAC^55^.

CDK7, XPB, and BRD4 are components of the basal transcription machinery. The fact that inhibitors of these proteins all exhibit SE-disruptive activity and consequently, antitumor activity, indicates that differences in the transcriptional control mechanisms (SEs in this case) between normal cells and disease-associated cells (e.g., cancer cells and the activated fibroblasts) can be exploited therapeutically. The selectivity of such therapeutic agents against normal and different disease cell types lies in the differences in SE activity. As illustrated in Fig. 3d, the genes affected by a SE-disruptive agent were dictated by whether or not the SEs associated with those genes were active. For example, in the MIA PaCa-2 cancer cells the majority of active SEs were associated with oncogenic genes such as MYC, whereas in CW5 CAFs cells, many SEs associated with genes related to fibrosis. Disruption of SEs may therefore selectively affect MYC and other oncogenic genes in cancer cells and genes regulating fibrosis in CAFs.

In summary, we provide compelling evidence that pancreatic CAFs and tumor cells harbor distinct SE networks that are characteristic of their different cell types and states. Based on the disruption of active chromatin marks and concomitant downregulation of SE-associated genes, triptolide might exert its antitumor activity by targeting distinct SE networks in different cells. We propose that epigenetic reprogramming of transcription by exploiting SE modulating compounds like triptolide alone or in combination with the current standard of care may provide more effective treatment options for patients with pancreatic cancer.

## Supplementary information

Supplementary Information (methods)

Figure S1, S2, S3

Table S1

Table S2

## References

[1] Siegel RL, Miller KD, Jemal A (2020). Cancer statistics, 2020. CA Cancer J. Clin..

[2] Rahib L (2014). Projecting cancer incidence and deaths to 2030: the unexpected burden of thyroid, liver, and pancreas cancers in the United States. Cancer Res..

[3] Garrido-Laguna I, Hidalgo M (2015). Pancreatic cancer: from state-of-the-art treatments to promising novel therapies. Nat. Rev. Clin. Oncol..

[4] Whatcott C, Han H, Posner RG, Von Hoff DD (2013). Tumor-stromal interactions in pancreatic cancer. Crit. Rev. Oncog..

[5] Erkan M (2012). The role of stroma in pancreatic cancer: diagnostic and therapeutic implications. Nat. Rev. Gastroenterol. Hepatol..

[6] Bailey P (2016). Genomic analyses identify molecular subtypes of pancreatic cancer. Nature.

[7] Evan GI (2017). Re-engineering the pancreas tumor microenvironment: a “regenerative program” hacked. Clin. Cancer Res..

[8] Hnisz D (2013). Super-enhancers in the control of cell identity and disease. Cell.

[9] Whyte WA (2013). Master transcription factors and mediator establish super-enhancers at key cell identity genes. Cell.

[10] Niederriter AR, Varshney A, Parker SC, Martin DM (2015). Super enhancers in cancers, complex disease, and developmental disorders. Genes.

[11] Sengupta S, George RE (2017). Super-enhancer-driven transcriptional dependencies in cancer. Trends Cancer.

[12] Chipumuro E (2014). CDK7 inhibition suppresses super-enhancer-linked oncogenic transcription in MYCN-driven cancer. Cell.

[13] Christensen CL (2014). Targeting transcriptional addictions in small cell lung cancer with a covalent CDK7 inhibitor. Cancer Cell.

[14] Kwiatkowski N (2014). Targeting transcription regulation in cancer with a covalent CDK7 inhibitor. Nature.

[15] Wu PP (2011). Triptolide induces apoptosis in human adrenal cancer NCI-H295 cells through a mitochondrial-dependent pathway. Oncol. Rep..

[16] Clawson KA, Borja-Cacho D, Antonoff MB, Saluja AK, Vickers SM (2010). Triptolide and TRAIL combination enhances apoptosis in cholangiocarcinoma. J. Surg. Res.

[17] Giri B (2019). Pre-clinical evaluation of Minnelide as a therapy for acute myeloid leukemia. J. Transl. Med..

[18] Borja-Cacho D (2010). TRAIL and triptolide: an effective combination that induces apoptosis in pancreatic cancer cells. J. Gastrointest. Surg..

[19] Chugh R (2012). A preclinical evaluation of Minnelide as a therapeutic agent against pancreatic cancer. Sci. Transl. Med..

[20] Zhao X (2020). Triptolide inhibits pancreatic cancer cell proliferation and migration via down-regulating PLAU based on network pharmacology of *Tripterygium wilfordii* Hook F. Eur. J. Pharm..

[21] Greeno E (2015). Phase I dose escalation and pharmokinetic study of 14-O-phosphonooxymethyltriptolide. Cancer Res..

[22] Banerjee S, Saluja A (2015). Minnelide, a novel drug for pancreatic and liver cancer. Pancreatology.

[23] Noel P (2019). Triptolide and its derivatives as cancer therapies. Trends Pharm. Sci..

[24] McGinn O (2017). Inhibition of hypoxic response decreases stemness and reduces tumorigenic signaling due to impaired assembly of HIF1 transcription complex in pancreatic cancer. Sci. Rep..

[25] Giri B (2017). “Heat shock protein 70 in pancreatic diseases: friend or foe”. J. Surg. Oncol..

[26] Garg B (2017). Modulation of post-translational modifications in beta-catenin and LRP6 inhibits Wnt signaling pathway in pancreatic cancer. Cancer Lett..

[27] Nomura A (2016). Inhibition of NF-kappa B pathway leads to deregulation of epithelial-mesenchymal transition and neural invasion in pancreatic cancer. Lab. Invest..

[28] Nomura A (2015). Minnelide effectively eliminates CD133(+) side population in pancreatic cancer. Mol. Cancer.

[29] Modi S (2016). Minnelide overcomes oxaliplatin resistance by downregulating the DNA repair pathway in pancreatic cancer. J. Gastrointest. Surg..

[30] Banerjee S (2016). Impaired synthesis of stromal components in response to Minnelide improves vascular function, drug delivery, and survival in pancreatic cancer. Clin. Cancer Res..

[31] Titov DV (2011). XPB, a subunit of TFIIH, is a target of the natural product triptolide. Nat. Chem. Biol..

[32] He QL (2015). Covalent modification of a cysteine residue in the XPB subunit of the general transcription factor TFIIH through single epoxide cleavage of the transcription inhibitor triptolide. Angew. Chem. Int Ed. Engl..

[33] Zhang Y (2008). Model-based analysis of ChIP-Seq (MACS). Genome Biol..

[34] Loven J (2013). Selective inhibition of tumor oncogenes by disruption of super-enhancers. Cell.

[35] Heinz S (2010). Simple combinations of lineage-determining transcription factors prime cis-regulatory elements required for macrophage and B cell identities. Mol. Cell.

[36] Jin Y (2018). Active enhancer and chromatin accessibility landscapes chart the regulatory network of primary multiple myeloma. Blood.

[37] Love MI, Huber W, Anders S (2014). Moderated estimation of fold change and dispersion for RNA-seq data with DESeq2. Genome Biol..

[38] Hingorani SR (2005). Trp53R172H and KrasG12D cooperate to promote chromosomal instability and widely metastatic pancreatic ductal adenocarcinoma in mice. Cancer Cell.

[39] Sugar E, Pascoe AJ, Azad N (2012). Reporting of preclinical tumor-graft cancer therapeutic studies. Cancer Biol. Ther..

[40] Pott S, Lieb JD (2015). What are super-enhancers?. Nat. Genet..

[41] Barrett MT (2017). Clinical study of genomic drivers in pancreatic ductal adenocarcinoma. Br. J. Cancer.

[42] Vareed SK (2011). Metabolites of purine nucleoside phosphorylase (NP) in serum have the potential to delineate pancreatic adenocarcinoma. PLoS ONE.

[43] Sodir NM (2020). Myc instructs and maintains pancreatic adenocarcinoma phenotype. Cancer Discov..

[44] Froeling FE (2009). Organotypic culture model of pancreatic cancer demonstrates that stromal cells modulate E-cadherin, beta-catenin, and Ezrin expression in tumor cells. Am. J. Pathol..

[45] Jameson GS (2019). Response rate following albumin-bound paclitaxel plus gemcitabine plus cisplatin treatment among patients with advanced pancreatic cancer: a phase 1b/2 pilot clinical trial. JAMA Oncol..

[46] Yinjun L, Jie J, Yungui W (2005). Triptolide inhibits transcription factor NF-kappaB and induces apoptosis of multiple myeloma cells. Leuk. Res..

[47] Lee KY, Park JS, Jee YK, Rosen GD (2002). Triptolide sensitizes lung cancer cells to TNF-related apoptosis-inducing ligand (TRAIL)-induced apoptosis by inhibition of NF-kappaB activation. Exp. Mol. Med..

[48] Dang CV (2012). MYC on the path to cancer. Cell.

[49] Hessmann E, Schneider G, Ellenrieder V, Siveke JT (2016). MYC in pancreatic cancer: novel mechanistic insights and their translation into therapeutic strategies. Oncogene.

[50] Buchholz M (2006). Overexpression of c-myc in pancreatic cancer caused by ectopic activation of NFATc1 and the Ca^2+^/calcineurin signaling pathway. EMBO J..

[51] Skoudy A, Hernandez-Munoz I, Navarro P (2011). Pancreatic ductal adenocarcinoma and transcription factors: role of c-Myc. J. Gastrointest. Cancer.

[52] Sancho P (2015). MYC/PGC-1alpha balance determines the metabolic phenotype and plasticity of pancreatic cancer stem cells. Cell Metab..

[53] Wirth M, Mahboobi S, Kramer OH, Schneider G (2016). Concepts to target MYC in pancreatic cancer. Mol. Cancer Ther..

[54] Delmore JE (2011). BET bromodomain inhibition as a therapeutic strategy to target c-Myc. Cell.

[55] Beglyarova N (2016). Screening of conditionally reprogrammed patient-derived carcinoma cells identifies ERCC3-MYC interactions as a target in pancreatic cancer. Clin. Cancer Res..

