# Supplementary material for: Triptolide targets super-enhancer networks in pancreatic cancer cells and cancer-associated fibroblasts
Source: Oncogenesis. 2020 Nov 9;9(11):100. doi: 10.1038/s41389-020-00285-9 (PMC7653036; doi:10.1038/s41389-020-00285-9)
Supplement: Supplementary file 1 — Supplementary Information (methods) [file 41389_2020_285_MOESM1_ESM.pdf]

Supplementary Methods

Cell culture and media

Pancreatic cancer cell lines MIA PaCa-2, PANC1, PSN1, AsPC1, BxPC-3, and Capan-1 were purchased from American Type Culture Collection (ATCC); PA-TU8988S and PA-TU8902 was purchased from German Collection of Microorganisms and Cell Cultures GmbH; and P4057 was established in-house from a patient's PDAC tumor tissues. All cancer cell lines were cultured in RPMI-1640 media supplemented with 10% FBS. Cancer associated fibroblast (CAF) cell lines, CW1, CW5, BF1, B010A, B010C, and B009B were isolated following published protocols (1) from resected PDAC patients' tumors under an Institutional Review Board (IRB) approved protocol. PS-1, a hTERT-immortalized human pancreatic stellate cell line was a gift from Dr. Hemant Kocher, Barts Cancer Institute, Queen Mary University of London (2). The CAFs and PS-1 cells were cultured in DMEM media containing 20% FBS. CAF08 cells are immortalized human pancreatic CAFs purchased from Neuromics (Minneapolis, MN, USA) and were cultured in MSCGro media supplied by Neuromics. The CAF cells were confirmed to express myofibroblast markers such as vimentin, alpha smooth muscle actin (αSMA), and Collagen I and were negative for epithelial markers such as EpCAM and cytokeratin using immunofluorescence staining. Cell lines were authenticated using short tandem repeat (STR) profiling at 4 month intervals during passage.

Triptolide was purchased from Tocris Bioscience (Cat # 3253), a Bio-Techne Company (Minneapolis, MN, USA). Minnelide was provided by Minneamrita Therapeutics LLC (Moline, IL, USA).

Chromatin immunoprecipitation

For non-drug treatment experiments, tumor cells or CAFs were plated in 60 mm tissue culture dishes and cultured for 48 hours before harvest. For drug treatment, cells were plated and culture overnight. Next day, TPL (20nM) or DMSO control was added to the cells and incubated for 18 hours. To harvest the cells, cell culture media was removed by aspiration and the cells were washed with phosphate buffered saline (PBS, pH 7.4). Cells were fixed and subjected to nuclei isolation using the truChIP Chromatin Shearing Reagent kit from Covaris Inc. (Woburn, MA, US) following manufacturer recommended protocol. Chromatin was sheared using a QSonica (Newtown, CT, USA) probe sonicator (10 seconds on; 30 seconds off; 25% amplitude for 18 cycles). Chromatin precipitation was carried out using the Novex Protein A Dyna Beads (Life Technologies) using either anti-Histone H3K27Ac antibody (Cat. #39133, Active Motif, Carlsbad, CA, USA) or anti-BRD4 (Cat. # 39909, Active Motif). The ChIP DNA was purified by the QIAquick PCR purification kit (QIAgen, Hilden, Germany) and quantified by a Qubit Fluorometer. The ChIP DNA input was also run on Agilent 4200 Tape Station to check for DNA quality.

Next generation sequencing

Ten nanograms or more of the ChIP DNA fragments were used to prepare sequencing libraries using an Illumina sequencing library preparation kit and QC was carried out using a Bioanalyzer (Agilent) (I think we need to write the model). Libraries with an average size of 200-300 bp were gel size selected and ethanol precipitation was then performed. The final libraries were sequenced using Illumina® (San Diego, CA, USA) sequencing platforms (HiSeq™ 2000 or NextSeq® 500).

Immunoblotting

Standard methods for immunoblotting were employed. Cells were lysed using Pierce™ IP Lysis Buffer (ThermoFisher Scientific, Waltham, MA, USA) with 1x concentration of Halt Protease & Phosphatase Inhibitor Cocktail (ThermoFisher Scientific). The solution was incubated on ice for 30 minutes before centrifugation at 13,000 g for 12 minutes at 4° to collect the lysate. Protein amounts in lysates were quantified using the Pierce BCA™ Protein Assay Kit (ThermoFisher Scientific). For each sample, 20 micrograms of total protein were loaded onto a NuPAGE™ 4-12% Bis-Tris Gel (Invitrogen). Ponceau Red was used to assess the quality of protein loading and transfer. The nitrocellulose blots were washed using standard methods utilizing 2% TBST Milk and TBST, and ultimately imaged on BioSpectrum Multispacial Imaging System (UVP).

Cell growth assay

A cell growth assay was conducted to assess the IC_{50} value of triptolide in inhibiting cell growth by measuring the total protein content within the cells using sulforhodamine B (SRB). 3,000 cells were seeded in each well of a 96-well plate and incubated overnight. Cells were then treated with two-fold dilutions of triptolide starting at a 1 micromolar concentration in triplicates and incubated for a total of 72 hrs. Following standard protocols for SRB staining (3, 4), the plates were read at 570 nm using the Synergy HT (Bio-TEK, Winooski, VT 05404).

RNA extraction and whole transcriptome RNA sequencing (RNA-seq)

The RNA-seq analysis was performed as previously described (5). RNA extraction was performed using the RNeasy® Midi Kit (Qiagen) using protocols recommended by the kit manufacturer. RNA sequencing libraries were constructed using the NEB Next® Ultra™ RNA Library Prep Kit (New England Biolabs) by Novogene. The concentration of libraries for RNA-seq from total RNA was first quantified using a Qubit 2.0 fluorometer (Life Technologies), and then diluted to 1 ng/µl before checking insert size on an Agilent 2100 and quantifying to greater accuracy by quantitative PCR (Q-PCR) (library activity >2 nM). Libraries were sequenced on an Illumina® HiSeq2500 system.

Immunohistochemical staining and scoring

Tumor tissues from vehicle or drug treated mice were harvested, formalin-fixed, and paraffin-embedded using standard procedures. Immunostaining of the mouse tumor sections (5µm) was carried out using a BondMax™ autostainer and the Bond™ Polymer Refine Detection kit (Leica Microsystems, Bannockburn, IL, USA) or MACH 3 Rabbit HRP Polymer Detection kit (Biocare Medical, Pacheco, CA). Heat-induced antigen retrieval was performed with a citrate based epitope retrieval solution (pH 6.0, Leica Microsystems). The dilution and incubation time for the primary antibodies used were: BRD4 (Abcam, Cat# ab128874), 1:100 dilution for 30 minutes; HSP70 (Abcam, Cat# ab181606), 1:400 for 30 minutes; MYC (Abcam, Cat# ab32072), 1:250 for 30 minutes; αSMA (Abcam, Cat# ab7817), 1:150 for 30 minutes; and Collagen 1 (COL1) (Novus Biologicals, Cat# NB600-408), 1:50 for 30 minutes. The secondary antibodies for the HRP polymer detection were provided as a part of the detection kits. The stained slides were scanned

and visualized using an Aperio Digital Pathology Slide Scanner (Leica). To evaluated the staining signals, staining intensity was scored as 0 (negative), 1 (weak), 2 (moderate), and 3 (strong). The number of positive cells (positive area for αSMA) was also scored as a percentage of the total cells (area). A combination score was then calculated by multiply the staining intensity score and the percentage positivity, for a maximum possible score of 300. This combination score was used to compare the marker difference between tumor tissues from vehicle and TPL treated mice.

Animal studies

All animal studies were carried out adhering to recommendations in the NIH Guide for the Care and Use of Laboratory Animals. The protocols were approved by the Institutional Animal Care and Use Committee (IACUC) at the University of Arizona, where the animal studies were carried out.

Transgenic KPC (LSL-KrasG12D/+; LSL-Trp53R172H/+; Pdx-1-Cre) mice were obtained based on the breeding scheme described by Hingorani and colleagues using 3 mouse strains, LSL-Trp53R172H/+, LSL-KrasG12D/+, and Pdx-1-Cre, which were obtained from National Cancer Institute Mouse Repository (6). Mice were fed *ad libitum* and housed at ambient temperatures (70–76°F). Tumor growth in the KPC mice was monitored using three-dimensional high resolution ultrasonography with the Visualsonics Vevo 770 system (Fujifilm Visualsonics, Ontario, Canada). Mice (both male and female) were enrolled into the study when the tumor size reached 120-200 mm^3. Mice (n=5) were treated with Minnelide via i.p. at 0.42 mg/kg daily for 7 days, after which tumor volumes were recorded and tumors were harvested. Total RNA was extracted from representative tumor pieces for RNA-sequencing (n=3) and immunostaining analyses (n=3).

The *in vivo* tumor growth inhibition activity of Minnelide alone or in combination with chemotherapy was evaluated using patient derived xenograft (PDX) models derived from two different PDAC patients (U01080713 and P4057). The PDX tumors were first established in athymic nude mice by subcutaneous flank implantation of tumor chunks taken directly from PDAC patients. Established tumors were propagated by implantation into a new set of female mice and allowed to grow to ~400 mm^3 in volume, at which point the mice were enrolled into the study. Once established, the tumors were subjected to genomic DNA sequencing analysis to verify that they retain the genetic alterations of the original patient tumor. A total of 4 treatment groups (7 mice/group) were included: 1) vehicle control (saline), 2) Minnelide, 3) nab-paclitaxel + gemcitabine + cisplatin, and 4) Minnelide + nab-paclitaxel + gemcitabine + cisplatin. Minnelide was administrated via i.p. at 0.42 mg/kg, daily for 21 days). Nab-paclitaxel was administrated via i.v. at 25 mg/kg. Gemcitabine was given i.p. at 70 mg/kg. Cisplatin was administrated via i.p. at 4mg/kg. Nab-paclitaxel, gemcitabine, and cisplatin were all given once a week for 3 weeks (on Days 1, 8 and 15). Mice were monitored for tumor growth twice weekly by measuring the greatest longitudinal diameter (length) and the greatest transverse diameter (width) of tumor using a caliper. Tumor volume was calculated by the formula: $(\text{length} \times \text{width}^2)/2$. Mice were euthanized when the tumor volume reached 2,000 mm^3.

GREAT analysis

SE regions that were identified in at least 3 of the cancer cell lines or CAF lines were selected to undergo genomic region annotation enrichment analysis using the GREAT (Genomic Regions

Enrichment of Annotations Tool) (7). The SE regions were uploaded to the GREAT website (http://great.stanford.edu) and analyzed using the default settings.

References

1. Bachem MG, Schneider E, Gross H, Weidenbach H, Schmid RM, Menke A, et al. Identification, culture, and characterization of pancreatic stellate cells in rats and humans. Gastroenterology. 1998;115(2):421-32.
2. Froeling FE, Mirza TA, Feakins RM, Seedhar A, Elia G, Hart IR, et al. Organotypic culture model of pancreatic cancer demonstrates that stromal cells modulate E-cadherin, beta-catenin, and Ezrin expression in tumor cells. Am J Pathol. 2009;175(2):636-48.
3. Skehan P, Storeng R, Scudiero D, Monks A, McMahon J, Vistica D, et al. New colorimetric cytotoxicity assay for anticancer-drug screening. J Natl Cancer Inst. 1990;82(13):1107-12.
4. Diep CH, Munoz RM, Choudhary A, Von Hoff DD, Han H. Synergistic effect between erlotinib and MEK inhibitors in KRAS wild-type human pancreatic cancer cells. Clin Cancer Res. 2011;17(9):2744-56.
5. Jin Y, Chen K, De Paepe A, Hellqvist E, Krstic AD, Metang L, et al. Active enhancer and chromatin accessibility landscapes chart the regulatory network of primary multiple myeloma. Blood. 2018;131(19):2138-50.
6. Hingorani SR, Wang L, Multani AS, Combs C, Deramaudt TB, Hruban RH, et al. Trp53R172H and KrasG12D cooperate to promote chromosomal instability and widely metastatic pancreatic ductal adenocarcinoma in mice. Cancer Cell. 2005;7(5):469-83.

7. McLean CY, Bristor D, Hiller M, Clarke SL, Schaar BT, Lowe CB, et al. GREAT improves functional interpretation of cis-regulatory regions. Nat Biotechnol. 2010;28(5):495-501.
