# Supplementary material for: Triptolide targets super-enhancer networks in pancreatic cancer cells and cancer-associated fibroblasts
Source: Oncogenesis. 2020 Nov 9;9(11):100. doi: 10.1038/s41389-020-00285-9 (PMC7653036; doi:10.1038/s41389-020-00285-9)
Supplement: Supplementary file 2 — Figure S1, S2, S3 [file 41389_2020_285_MOESM2_ESM.pdf]

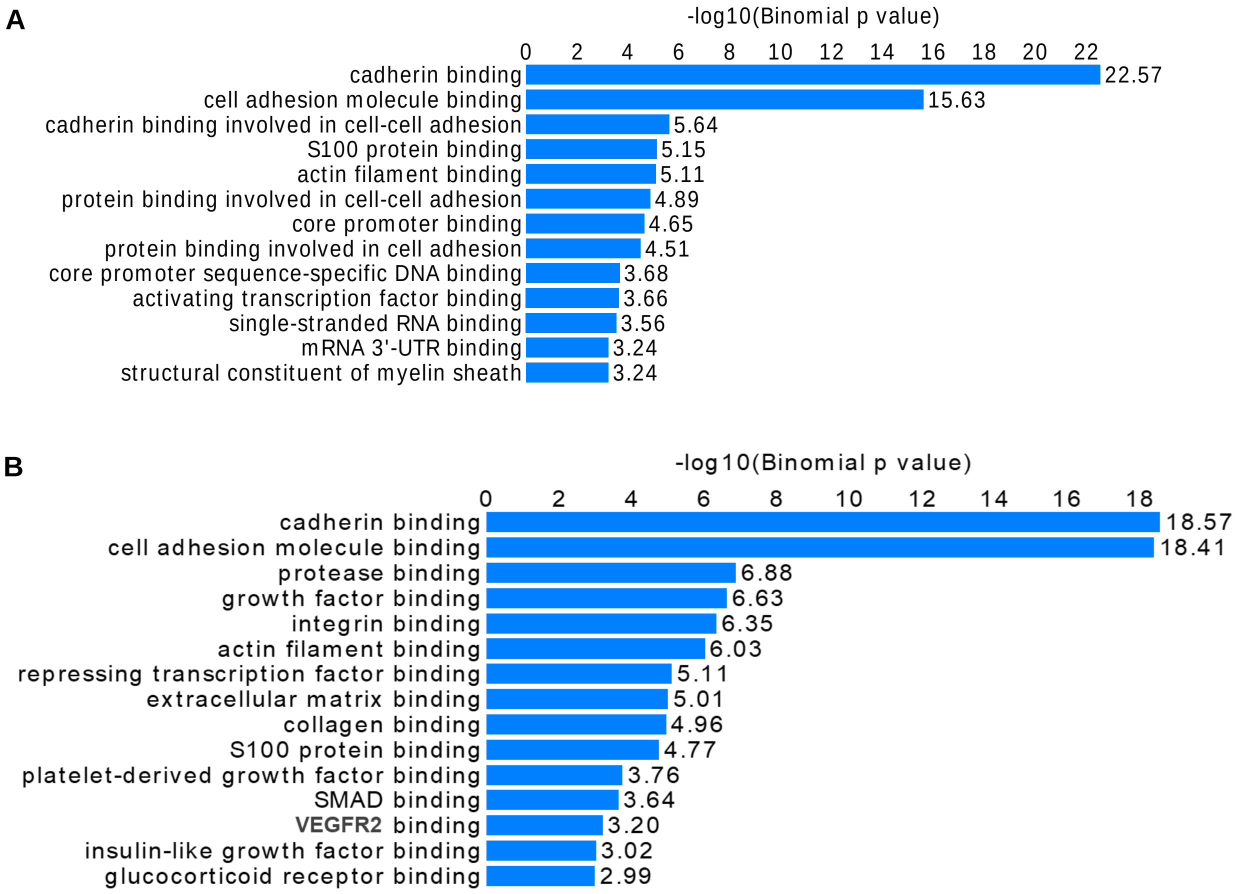

Supplementary Figure S1: Gene Ontology (GO) molecular function terms enriched in super-enhancer regions in PDAC cancer cell lines (A) and CAF cell lines (B).

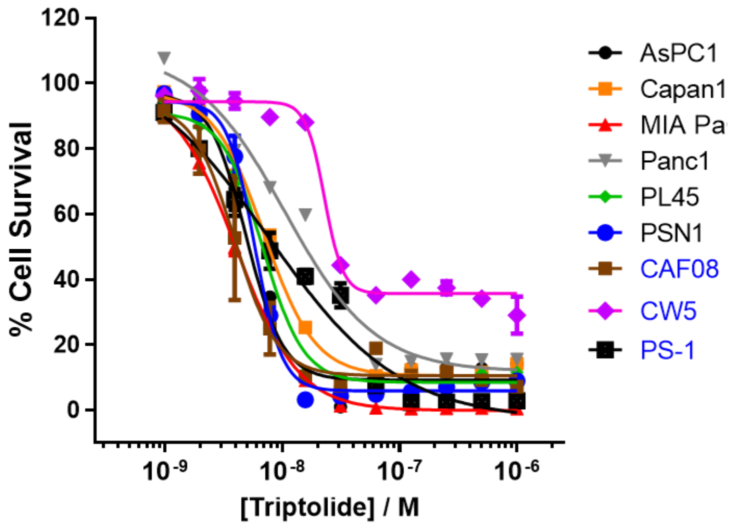

Supplementary Figure S2: Dose response curves of triptolide in a panel of pancreatic cancer cell lines (black text) and activated fibroblasts (blue text). Cells were treated with a serial dilution triptolide (1nM to 1 µM) for 72 hours.

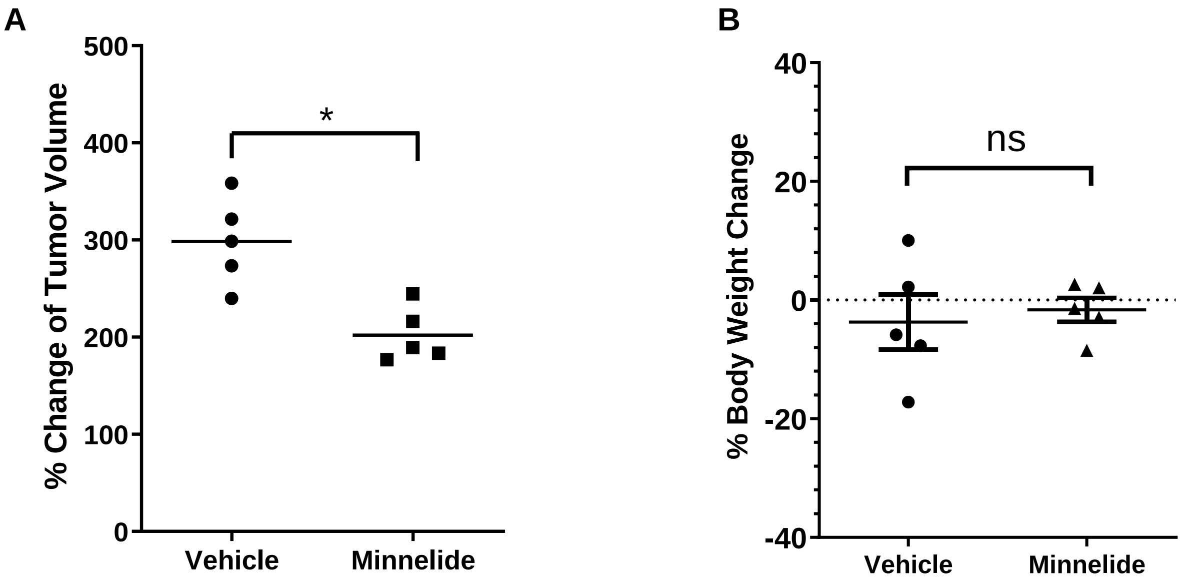

Supplementary Figure S3: Minnelide treatment significantly reduced tumor growth in the KPC genetically engineered mouse model. A) Tumor volume changes before and after treatment. Mice (n=5) were treated with Minnelide at 0.42 mg/kg, QD, i.p. for 7 days at which point the tumor volumes were recorded and compared to the pretreatment tumor volume. The tumor tissues were then harvested (3 mice/group) and used for biological analyses (see text for details). B) Mouse body weight changes before and after treatment. * P values < 0.004. ns: not significant.
